# RNA structural analysis of the *MYC* mRNA reveals conserved motifs that affect gene expression

**DOI:** 10.1371/journal.pone.0213758

**Published:** 2019-06-17

**Authors:** Collin A. O’Leary, Ryan J. Andrews, Van S. Tompkins, Jonathan L. Chen, Jessica L. Childs-Disney, Matthew D. Disney, Walter N. Moss

**Affiliations:** 1 Roy J. Carver Department of Biophysics, Biochemistry and Molecular Biology, Iowa State University, Ames, IA, United States of America; 2 Department of Chemistry, The Scripps Research Institute, Jupiter, FL, United States of America; Ben-Gurion University, ISRAEL

## Abstract

The *MYC* gene encodes a human transcription factor and proto-oncogene that is dysregulated in over half of all known cancers. To better understand potential post-transcriptional regulatory features affecting *MYC* expression, we analyzed secondary structures in the *MYC* mRNA using a program that is optimized for finding small locally-folded motifs with a high propensity for function. This was accomplished by calculating folding metrics across the *MYC* sequence using a sliding analysis window and generating unique consensus base pairing models weighted by their lower-than-random predicted folding energy. A series of 30 motifs were identified, primarily in the 5' and 3' untranslated regions, which show evidence of structural conservation and compensating mutations across vertebrate *MYC* homologs. This analysis was able to recapitulate known elements found within an internal ribosomal entry site, as well as discover a novel element in the 3' UTR that is unusually stable and conserved. This novel motif was shown to affect *MYC* expression, potentially via the modulation of miRNA target accessibility or other trans-regulatory factors. In addition to providing basic insights into mechanisms that regulate *MYC* expression, this study provides numerous, potentially druggable RNA targets for the *MYC* gene, which is considered “undruggable” at the protein level.

## Introduction

The *MYC* proto-oncogene is an important transcription factor that is required for programmed cell death (apoptosis) and cell proliferation [1]. It is a key component of oncogenesis [2] and, indeed, *MYC* is dysregulated in >50% of all cancers [3]. Post-transcriptional control plays significant roles in the regulation of many genes including MYC. Within the 5' untranslated region (UTR) of the *MYC* mRNA is a structured internal ribosomal entry site (IRES) that stimulates cap-independent translation under apoptotic conditions and other instances where cap-dependent translation is inhibited [4]. Consistent with many other IRESs [5] the *MYC* IRES secondary structure (deduced from *in vitro* chemical probing data [6]) contains pseudoknots, which are motifs comprised of “non-nested” base pairing between looped out regions of RNA [7]. In addition to the IRES, other post-transcriptional regulatory mechanisms affect *MYC* expression that may be affected by *MYC* RNA structure [8]: e.g. microRNAs (miRs) [9].

To determine if other structured RNA regulatory elements have roles in *MYC* expression, we applied a methodological pipeline for RNA motif discovery that was optimized from studies of the *Xist* lncRNA [10], as well as the Human [11], Zika and HIV genomes [12]. There are two major steps in this pipeline: (1) a scanning step, where the RNA is examined using a sliding analysis window to record predicted metrics important for analyzing RNA secondary structure (e.g. the thermodynamic stability) and (2) an analysis step where unique local motifs are defined then evaluated vs. comparative sequence/structure and/or experimental probing data. Each step is achieved using the programs ScanFold-Scan and ScanFold-Fold, respectively. Used together these programs define the potential RNA structural properties of long sequences and identify motifs likely to be ordered to form, presumably functional, defined secondary structures. This is accomplished by generating consensus structure models across all scanning windows, where base pairs are weighted by their thermodynamic z-score: a measure of the unusual stability of a sequence that is calculated by comparison to the folding energy of matched randomized control sequences. Here, negative values indicate sequences that are ordered to fold and that may be functional [13].

In this report, ScanFold-Scan and ScanFold-Fold were applied to the longest *MYC* RefSeq mRNA isoform to generate a map of its folding landscape as well as deduce motifs important to the regulation of expression. Numerous motifs were detected, including those that recapitulated known structures in the *MYC* IRES. One motif from the 3' UTR with exceptional predicted folding metrics was analyzed experimentally and found to play roles in the regulation of *MYC* expression.

## Results

### ScanFold-Scan mapping of secondary structure in the *MYC* mRNA

**Fig 1:**
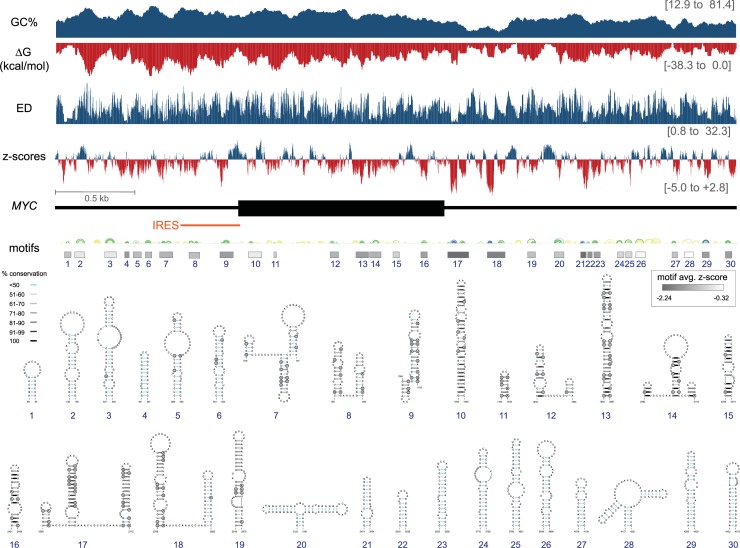
Summary of ScanFold-Scan and ScanFold-Fold results for the *MYC* mRNA. At the top are charts indicating the predicted ScanFold-Scan metrics across the mRNA. The bars are set at the 1^st^ nt of the 70 nt window, thus data corresponds to the 70 nt downstream of the bar. On the right, in brackets, the maximal and minimal values of each predicted metric for windows spanning the *MYC* mRNA are given. Below these charts is a cartoon of the *MYC* mRNA with UTRs and coding region represented in thin and thick black lines, respectively. This cartoon is annotated with boxes which depict the location and extent of ScanFold-Fold predicted motifs shaded based on the average z-score of windows in which motif base pairs occurred. Above these are RNA secondary structure arc diagrams which depict the most favorable base pairs predicted via ScanFold-Fold, colored according to the average z-scores of windows in which they appear (with blue, green and yellow corresponding to less than -2, -1 and 0 z-score averages respectively). Below these, are refolded models of the motifs built with -1 average z-score bp as constraints. Each is annotated with their bp conservation as determined from an alignment of 15 representative mRNAs (S3 File) indicated by shading on the base pair (see key). Circled bases are sites of putative structure-preserving consistent and compensatory mutations.

To predict RNA secondary structural characteristics important to *MYC* function, the RefSeq mRNA (NM_002467.5) was analyzed using the program ScanFold-Scan [12]. The mRNA sequence was analyzed using a 1 nt step and 70 nt window size (Fig 1; S1 File). Several folding metrics were calculated across analysis windows which are described in detail in the Materials and Methods and in reference [11]. Briefly, the ΔG° measures the minimum (lowest or most stable) predicted change in the Gibb’s free energy upon RNA folding and indicates the thermodynamic stability of RNA structure. The ensemble diversity (ED) is a measure of the structural diversity predicted in the folding ensemble; low numbers indicate one or few dominant structures, while higher numbers indicate multiple conformations or a lack of structure. The thermodynamic z-score measures the propensity of a sequence to be ordered to fold into stable structures. Negative z-scores give the number of standard deviations more thermodynamically stable a sequence is vs. random (see Eq 1 in Materials and Methods).

The global trends in each metric are shown at the top of Fig 1. The trend in the predicted thermodynamic stability approximately follows the GC% and decreases across *MYC*: going from the highly stable 5' UTR to the relatively unstable 3' UTR. ED values are more uniformly distributed across *MYC*, however, jumps in the mean ED value of windows spanning the 5' and 3' UTR coding region junctions were observed (Fig 1). Thermodynamic z-scores ranged from highly negative (-5.0; or 5 standard deviations more stable than random) values to positive ones (+2.8). The average z-score across *MYC* was only slightly negative (-0.4) and there was no evidence of global bias in z-score toward negative values. In general, the z-score and ED metrics appear independent of thermodynamic stability; both metrics only weakly correlate to ΔG° with 0.396 and 0.067 correlation coefficients with z-score and ED respectively (S1 Table). To determine if, on average, certain regions have propensity for relatively higher or lower metrics, each region of the *MYC* mRNA was regarded as its own discrete segment: 5' UTR, coding region, 3' UTR, and the junctions between them. Box and whisker plots depicting the distributions of metrics for each region are shown in S1 Fig. Unsurprisingly, the ΔG° values for each region follow the global trend (becoming less stable towards the 3' end) however, the trends for the ED and z-score metrics appear to correspond more to location than underlying ΔG° values. The highest mean ED values (most diverse folding ensembles) were observed for the 5' and 3' junctions (15.17 and 14.81 respectively; S2 Table). Despite being almost 1000 nt apart, the high ED values for these regions were the most similar when mean values from each region were compared against all others while the only other regions to display comparable similarity of their mean ED values were the UTRs, which had the *lowest* mean ensemble diversity values (S2 Table). Similar trends were observed for the mean z-scores (which is expected considering the slight correlation between ED and z-scores overall (S2 Table). The mean z-scores for the 5' and 3' junctions were the highest (-0.17 and -0.08), while the mean z-scores for the 5' and 3' UTRs were the *lowest* (-0.54 and -0.45). A statistical analysis was attempted to assess the significance of these similarities (using a two-sample t-test assuming unequal variance; S3 Table) however it is important to note that interpretations of these p-values are limited by the fact that the underlying assumption of independence required for the t-test does not hold for scanning window analyses.

### *ScanFold-Fold* prediction of functional RNA structural motifs

To deduce local RNA folding that may be functionally significant, all ScanFold-Scan prediction windows were analyzed using ScanFold-Fold. The ScanFold-Fold program generates weighted consensus secondary structures, where minimum free energy (MFE) base pairs that contribute to low z-scores are deduced across the scans. Using a cutoff of z-score < -1 ScanFold-Fold identified 354 bp (S2 File) across the mRNA, while a cutoff of z-score < -2 yields 46 bp that are localized to the 3' UTR. Refolding the mRNA with z-score < -1 ScanFold-Fold bp as constraints added 153 bp to the discovered motifs by extending helices or closing unpaired bases in the consensus prediction (S2 File). These 507 bp are divided into 30 motifs that span the *MYC* mRNA (Fig 1). Motif locations, as expected, correspond to negative dips in z-score; however, dips in ΔG° and ED are also observed at motif sites. The most prominent regions with favorable dips in metrics occur at Motifs 17 and 18 (Fig 1), which contain very low z-score base pairs deduced by ScanFold-Fold (z-score cutoff < -2). These two motifs, particularly Motif 17, had the most favorable ScanFold metrics of any region/motif predicted for the *MYC* mRNA.

A notable feature of the resulting 2D structural motifs is the presence of multiple RNA tetraloops with interesting sequence composition. RNA tetraloops play important structural and functional roles in many known structured RNAs [14]: e.g. approximately 55% of RNA helices in the *E*. *coli* 16S rRNA are capped by tetraloops [15]. There are 12 tetraloops predicted to form in predicted structured regions of the *MYC* mRNA, which fall within 9 predicted structural: Motifs 4, 9–12, 14, 16,17, 20, 25, and 28 (Fig 1). Several of these tetraloops are representatives of large families of tetraloops: e.g. Motif 11 contains a UNCG tetraloop [16]; Motif 16 has an RNYA tetraloop [17]; and Motif 28 contains a GNRA tetraloop [15, 18]. Additionally, considering the possibility of stabilizing purine-purine “sheared” pairs, several other tetraloops are possible. For example, in the 5' hairpin of Motif 17 an RNYA tetraloop is possible above a GA sheared pair (Fig 1).

To compare local motif structure predictions to a global model of mRNA folding, an unconstrained energy minimization prediction was performed for *MYC* (S2 File). 57% of the base pairs in the 30 motifs also appeared in the unconstrained global secondary structure model and 8 of the 30 motifs were outcompeted completely by alternative longer-range pairing (S4 Table). Motifs 17 and 18, however, had 91 and 100% of their bp predicted in the global model, indicating little propensity for competing interactions. In Motif 17, for example, three base pairs in the basal portion of the 3' hairpin are broken to form a small hairpin comprised of two CG pairs in the global model (S2 File). These alternative bp are not scored well by ScanFold-Fold and are not well-supported by the *MYC* sequence alignment (S3 File).

All motif bp were analyzed versus an alignment of 15 vertebrate mRNA sequences (S3 File). Motif 17 had the highest conservation of structure and was supported by the greatest number of consistent and compensatory mutations (Fig 1). In general, Motifs 7–19 showed evidence of conservation, however, little conservation data was found outside these regions: particularly downstream of Motif 19, where the long 3' UTR annotated for the human *MYC* RefSeq mRNA is not present in the RefSeq mRNA annotations of other species (S3 File).

### Analysis of the *MYC* 5' and 3' UTRs

Motifs 8 and 9 overlap a previously-studied IRES in the *MYC* mRNA [4, 6]. Motif 8 is recapitulated in the *MYC* IRES structure Domain 1; only the base pairs in the hairpin spanning nt 110 to 136 (S2 Fig) are shifted over to allow the formation of pseudoknot helix α. Motif 9 partially overlaps Domain 2, where nt 284 to 299 of Domain 2 are refolded into two hairpins (S2 Fig). Structure models were compared vs. an alignment of 50 vertebrate *MYC* UTR sequences (S4 File). The alternative models for Domain 2 (Motif 9) are roughly equally well supported by comparative data. Both are comprised of base pairs conserved across vertebrates and show evidence of possible compensatory mutations: e.g. C279–G284 in Domain 2 vs. A307–U334 and G309–C332 in Motif 9 (S2 Fig). Neither model can be discarded based on these data. Nucleotides within Motif 9 were found to be highly reactive to chemicals in the previous *in vitro* analysis of the *MYC* 5' UTR [6], thus their modeling as single stranded RNA. When overlaid on Motif 9, however, only 4 out of 21 chemical modification sites (DMS, kethoxal and CMCT; probes of single-stranded RNA) were inconsistent with the ScanFold-Fold generated model (S2 Fig); additionally, sites of AMV reverse transcriptase pausing suggest that this region is structured.

**Fig 2:**
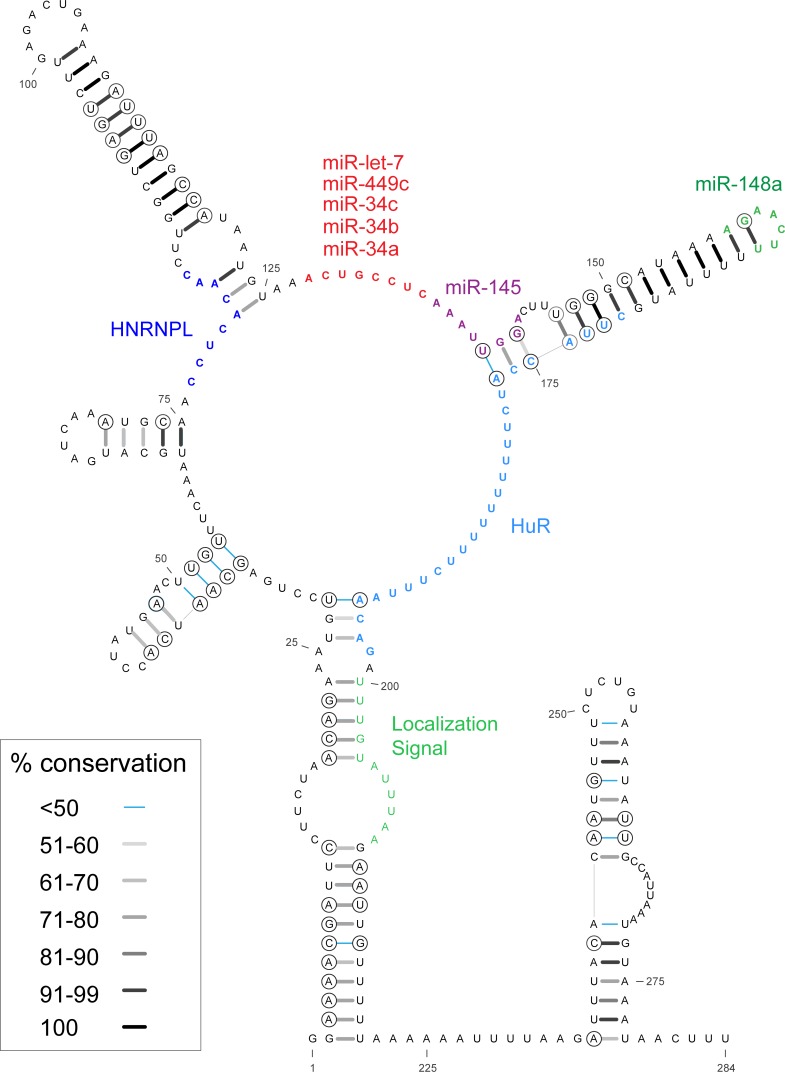
Short *MYC* 3' UTR model. Nucleotides 60–177 comprise Motif 17 of the ScanFold-Fold results, which were constrained in the calculation. Base pair conservation shading indicated in the key and data are taken from a comparison of 59 RefSeq mRNA vertebrate alignment (S5 File). Circled bases are sites of putative structure-preserving consistent and compensatory mutations. We have also annotated the sequence with relevant functional motifs: miRNA binding site seed sequences are highlighted (red, purple and green); RBPMap predictions for HNRNPL’s binding sites are colored blue (S7 File; validated by CLIP-seq data [30]); the 28 nt HuR binding site (as reported in [28]) is colored in light blue; and a highly conserved region shown to be a localization signal as reported in [37] is colored in a light green.

Across the *MYC* mRNA, predicted structural metrics are most favorable in the windows that overlap Motif 17 in the 3' UTR (Fig 1). There are marked dips in the ΔG°, ED and z-score; all indicating importance of structure in this region. The ScanFold-Fold predicted base pairs in Motif 17 are also the best-conserved across the 15 vertebrate alignment. Previous work on post-transcriptional regulation of *MYC* found that inclusion of the short 3' UTR sequence led to repression of luciferase expression [19] due to the inclusion of a miR-34 binding site. To determine if RNA structural features in the short 3' UTR (beyond Motif 17) could be playing additional roles, the entire sequence was refolded while constraining Motif 17 base pairs. The resulting global short UTR model (Fig 2) places the ScanFold-Fold predicted Motif 17 into a multibranch loop structure that includes an additional short hairpin. Another hairpin is also predicted downstream of the multibranch loop. The short 3' UTR model was analyzed vs. an alignment of 59 vertebrate *MYC* 3' UTR sequences (S5 File). This found the highest levels of base pair conservation in the two long Motif 17 hairpins (92% conservation), while the remaining structures are not well-conserved (64% conservation of base pairing). When mutations occur in the highly-conserved Motif 17 they preserve base pairing: e.g. four compensatory (double point) mutations are found in each hairpin in addition to four and two consistent (single point) mutations, respectively (Fig 2). To see if an orthogonal approach would confirm the 3' UTR model structure or, perhaps, yield a better-conserved alternative model, the program RNAalifold [20] was used to evaluate the short 3' UTR alignment without any base pairing constraints. The RNAalifold program considers both the folding energy and comparative sequence data (implicitly) in prediction; the resulting consensus model (S3 Fig) predicts conserved structures that correspond to the two highly-conserved Motif 17 hairpins predicted by ScanFold-Fold.

### Functional analyses of the *MYC* 3' UTR

As the most significant motifs predicted in *MYC* occurred in the 3' UTR, a known site of miRNA targeting, the locations of *MYC*-targeting miRNA binding sites were queried vs. predictions of structure. Of nine miRNAs with known interaction sites [9, 19, 21–25], *seven* occurred within Motif 17 (Fig 2; S4A and S4B Fig). miR-34a/b/c, miR-449c and let-7a have overlapping seed binding sites in the unstructured region between the two highly-conserved Motif 17 hairpins (Fig 2; S4A and S4B Fig). miR-145 binds downstream and partially overlaps the second hairpin. miR-148 has a seed binding site on the terminal stem-loop of the second hairpin. Interestingly, the two miRNAs that bind outside Motif 17 also do so in other ScanFold-Fold predicted structural motifs: miR-24 binds in the stem region of Motif 18 (S4C Fig), while miR-185 binds toward the 5' end of Motif 15 (S4D Fig). In all cases, conserved RNA structures are predicted to partially occlude miRNA target binding, potentially modulating their effects.

**Fig 3:**
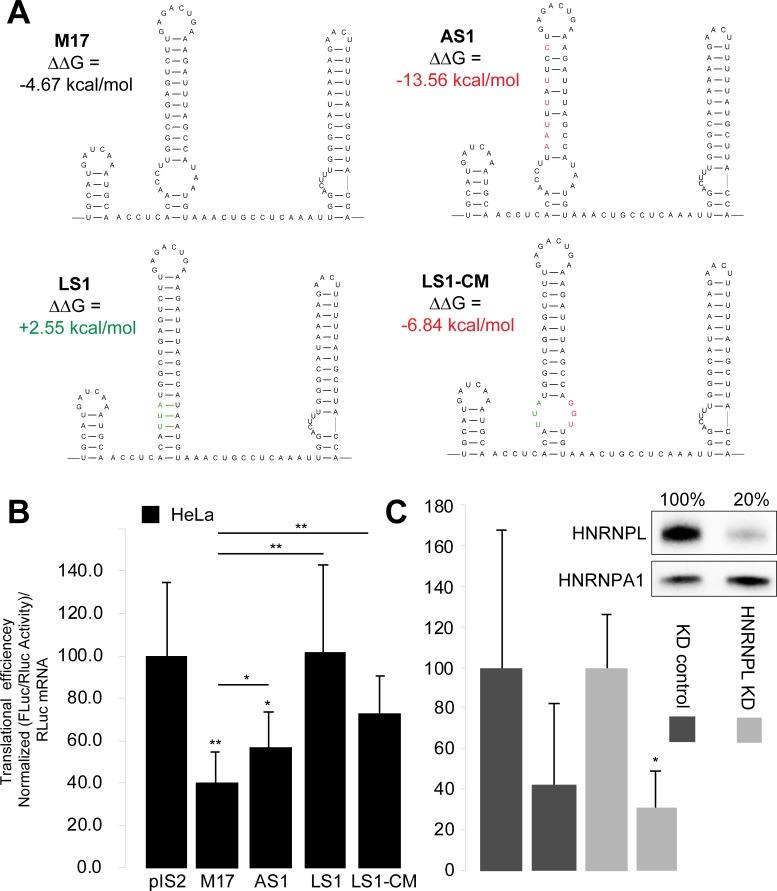
Structural models for Motif 17 constructs and their translation efficiencies. **A**) The predicted secondary structure for the wild type pIS2-M17 construct is shown, and mutations are depicted in context of this native secondary structure (within the figure, we have dropped the pIS2 notation from experimental constructs). Mutations which disrupt the native structure are depicted as red when decreasing ΔΔG and as green when increasing ΔΔG. In the case of LS1-CM, the original mutations are depicted in green and the compensatory mutations are in red. The ΔΔG values for each construct were calculated using PITA [26]. **B**) Several Motif 17 constructs were tested for their effect on TE of *Renilla* luciferase (pIS2). pIS2-M17 displays a statistically significant decrease in TE and pIS2-AS1 displays a TE which is significantly increased compared to pIS2-M17 but remains significantly decreased from pIS2 control. The TE of pIS2-LS1 is significantly greater than pIS2-M17, and while the pIS2-LS1-CM construct has a lower TE, it is not statistically different than either pIS2-LS1 or pIS2. **C**) pIS2-M17 was expressed in control and HNRNPL knockdown HeLa cells to test for differences in TE versus control (pIS2). In both cases, pIS2-M17 displayed a decrease in TE whereby the knockdown of HNRNPL under our conditions did not appear to have an effect on TE. All experiments (B and C) were run in at least triplicate; TEs were calculated for each set of samples and normalized to the unregulated activity of pIS2; error bars report the standard deviation; statistical significance for two sample unpaired *t-*tests assuming unequal variance are reported as asterisks (* for p < 0.05, ** for p < 0.005) and unless shown with a corresponding bar, are for comparison with the pIS2 control.

Motif 17 was selected for additional experimental analysis due to it having the strongest ScanFold prediction metrics (Fig 1), high level of structure conservation and the presence of multiple miRNA binding sites (Fig 2). To assess the potential gene regulatory roles of this motif, a luciferase reporter construct was generated incorporating Motif 17, along with 27 nt upstream and 11 nt downstream (including a poly(U) tract; nt 33–188 in Fig 2). This sequence was inserted into the 3' UTR of the *R**enilla* luciferase (RL) expressing pIS2 vector (Fig 3A, referred to as the pIS2-M17 [Motif 17] vector; detailed in the Materials and Methods). When assayed, the pIS2-M17 vector showed a significant decrease in translational efficiency (TE) when compared to the unregulated pIS2 control: a ~58% decrease (Fig 3B; S6 File). This is consistent with previous analyses of the *MYC* 3′ UTR, where the entire short UTR isoform was incorporated into an analogous luciferase vector (pLSV) and, using a similar analysis pipeline, was shown to lead to gene repression [19]. Similarly, ablation of the miR-34a-c seed (and also, seed regions for miRs 449c and let-7a) showed that miRNA targeting was responsible for the repressive effects of this region [19].

To determine if RNA structure present in Motif 17 influences miRNA binding/repression, two mutant constructs, pIS2-AS1 (ablate stem 1) and pIS2-LS1 (lock stem 1), were designed to increase or decrease miRNA site accessibility, respectively (AS1 and LS1 in Fig 3A), according to the ΔΔG metric of Kurtesz et. al [26]. This metric accounts for both the energy needed to break native mRNA secondary structure and the energy gained by miRNA binding and was used to predict miRNA site accessibility for the WT and mutant constructs. The WT sequence, pIS2-M17, has a predicted ΔΔG of -4.67, whereas pIS2-AS1 and pIS2-LS1 have values of -13.56 (more accessible) and +2.55 (less accessible) respectively. When assayed, pIS2-AS1 shows an increase in TE of ~20% compared to pIS2-M17 (Fig 3B). pIS2-LS1 showed a ~60% increase in TE when compared to pIS2-M17, yielding a TE that is statistically equivalent to the unregulated pIS2 control (Fig 3B).

To test if structural stability alone was responsible for the increase in TE, a “compensatory” mutant was made for LS1 (Fig 3A; pIS2-LS1-CM, detailed in Materials and Methods), which reintroduced a bulge (similar to that of pIS2-M17) by making mutations in the miRNA binding region, but outside of the seed-binding region. This construct results in a slightly more negative ΔΔG (-6.84 kcal/mol) than pIS2-M17 (-4.67 kcal/mol). When assayed, this compensatory mutant reduced the mean TE value (vs pIS2-LS1), but not to a degree that was statistically significant. This lack of rescue could indicate the presence of other factors, such as regulatory RNA binding proteins (RBPs), which may also be targeting Motif 17. For example, the RBP HuR was shown to be a necessary factor for let-7a targeting of *MYC*; this miRNA has a binding site within Motif 17 (Fig 2) [27, 28]. To assess if any other RBPs could be affecting the TE of Motif 17, its sequence was analyzed using RBPmap, which makes predictions based on primary sequence motif [29]. A total of five RBPs (HNRNPL, MATR3, MBNL1, SRSF3, and YBX2) were predicted to bind to at least one of the nucleotides mutated in pIS2-LS1 (nt 25–28 in S7 File). Recent studies using UV crosslinking and immunoprecipitation (CLIP) found that HNRNPL does indeed bind this region in several cell types [30, 31].

To assess if HNRNPL plays a role in the decreased TE observed for the pIS2-M17 luciferase construct, HeLa cells were transduced with an HNRNPL-targeting shRNA construct. HNRNPL knockdown (KD) was assessed via western blotting and showed a ~80% decrease in HNRNPL expression compared to cells transduced with non-targeting shRNA control (Fig 3C). Luciferase assays were repeated with pIS2 and pIS2-M17 in both the control and KD HeLa cells, yielding statistically similar decreases in TE (Fig 3C). This indicates that, under the conditions of our assay, HNRNPL does not appear to affect the TE and should not be affected by the mutations in pIS2-LS1.

## Discussion

The analyses performed in this report provide insights into the functions of RNA secondary structure in expression of *MYC*. The ScanFold-Scan results map out local features of RNA structure across the *MYC* mRNA. Interesting trends are observed moving across the sequence, where RNA thermodynamic stability decreases going 5' to 3', with marked “jumps” in instability observed at the UTR/coding-region junctions (S1 Fig; S1–S3 Tables). Likewise shifts toward more positive ED and z-score values were observed in junction-spanning windows: which could be of particular functional interest at the 5' junction, which includes both the CUG (non-canonical) and AUG (canonical) translation initiation sites. These results indicate a lack of stable structure here, reiterating previous observations that indicate inhibitory roles for thermodynamically stable RNA secondary structure at initiation sites [32]. We additionally find evidence that evolution may be specifically selecting for *MYC* initiation site sequences that are ordered to be less stable than that predicted for sequences of similar composition (thus the presence of several positive z-scores at these junctions; S1 File); as well, the junction sequence is expected to have a volatile conformational ensemble, where no particular structure dominates (high ED).

The high and low respective thermodynamic stabilities of the 5' and 3' UTRs (S1 Fig) indicate differing roles for RNA folding in these regions. The highly stable 5' UTR would be expected to inhibit canonical translation by obstructing scanning ribosomes; thus, the presence of an IRES in the *MYC* mRNA. This can provide a mechanism for fine-tuning the post-transcriptional regulation of the *MYC* gene, by regulating the amounts of cap-dependent vs. cap-independent translation. The *MYC* IRES was shown to be active in some, but not all tissue types and the variability of activity is attributed to the presence, or lack of, trans-regulatory elements (e.g. RBPs; [4]). This demonstrates how cis-elements of the mRNA can interact with trans-regulatory elements to diversify (i.e. regulate) the cellular levels of a protein.

In contrast, the low stability of the 3' UTR suggests a need for increased accessibility of the mRNA sequence: e.g. for intermolecular interactions with post-transcriptional regulatory factors such as miRNAs and regulatory proteins. Counterintuitively, the sites with the greatest evidence of having been ordered to fold into a specific structure are in the 3' UTR (e.g. Motifs 17 and 18 in Fig 1). Motif 17, for example, is the most well-conserved structured region in *MYC*—even more so than the IRES domain (Fig 2 and S3 Fig)—and is supported via multiple compensatory and consistent base mutations. The highly favorable metrics and deep conservation of this motif throughout vertebrates indicated its biological importance, which was borne out by the analysis of miRNA binding sites (Fig 2; S4A and S4B Fig) and Motif 17 function (Fig 3).

The second most favorable motif (Motif 18; Fig 1) contains a miR-24 interacting region (S4C Fig). Notably, this interaction is “seedless” [22]—only three of the miR-24 seed nt are base paired to *MYC* (S4C Fig). Most of the miR-24-interacting nt on *MYC* are predicted to be bound up in structure. Here, as in other interaction sites, RNA folding may be modulating accessibility. Additional functional motifs are predicted downstream of Motif 18 (Fig 1), which may also be functionally significant. Interestingly, cancer-associated *MYC* translocations [33] can lead to UTR truncations that delete predicted motifs: potentially impacting function and contributing to *MYC* dysregulation. Likewise, seven predicted motifs fall within the *MYC* coding region, which may be functionally significant: e.g. by providing roadblocks for translation that can affect protein folding [34] or by affecting interactions with regulatory factors [35]. Notably, miR-185 targets a sequence that overlaps Motif 15 (S4D Fig), which falls within the *MYC* coding region (Fig 1). Awareness of the importance of miRNA targeting in coding regions is growing [36] and, presumably, additional *MYC* miRNA interactors remain to be discovered.

Previously, three short (< 12 nt) regions in the *MYC* 3’UTR were highlighted as having ~100% sequence conservation across several species separated by over 350 million years of evolution [37]. Veyrune et. al. [37] proceeded to characterize one of these regions and found that it was a localization signal (nt 200–210 in Fig 2) responsible for localizing *MYC* mRNAs to the perinuclear cytoplasm. The two other hyper-conserved regions both span Motif 17: one encompasses the miR34a/b/c, miR449c and let-7a miRNA seed sequence regions (nt 127–135 in Fig 2), and another occurs toward the 5' end of the first conserved hairpin of Motif 17 (nt 80–91 in Fig 2). This latter motif contains the bases mutated in pIS2-LS1 which (when introduced) led to a dramatic increase in TE when compared to native Motif 17 (Fig 3B). The evidence provided by our functional analyses support the idea that this highly conserved region is necessary for the fine-tuned regulation of the *MYC* mRNA. One interpretation of this result is that mutations could be affecting hairpin stability; however, the compensatory pIS2-LS1-CM mutant that restored the internal loop by mutating bases on the 3' end of the hairpin, did not restore WT TE (Fig 3B). Interpretation of these results is complicated by the mutated “compensatory” bases occurring in a region that is overlapped by multiple miRNA binding sites. It is likely that the effects observed in the WT pIS2-M17 construct are due to a combination of factors: the structure/accessibility of Motif 17 and its multitude of potential interactions with trans-acting RBPs and miRNAs. More investigation is needed to parse out the exact mechanistic details of this functional motif.

To conclude, this report provides RNA secondary structural data across the *MYC* mRNA and identifies discreet local motifs with a high propensity for function. This includes a particularly interesting motif in the 3' UTR which is functionally important. Our findings illustrate the utility of ScanFold-Scan and ScanFold-Fold in finding structured, regulatory motifs and highlights the important role of RNA secondary structure in the post-transcriptional gene regulation of *MYC* expression. This study provides a roadmap for further analyses of the structure/function relationships in the *MYC* mRNA and a framework for understanding other experimental results. For example, identified clinically significant sequence variants can be cross-referenced to these results to deduce their potential impact on RNA folding. Additionally, these results generate a list of structural motifs that may be druggable targets [38, 39] for *MYC*, which is considered undruggable at the protein level [3].

## Materials and methods

### *In silico* analyses

The *Homo sapiens MYC* RefSeq mRNA sequence was downloaded from the NCBI nt database (GenBank Accession: NM_002467.5). ScanFold-Scan was run using a single nt step size and window sizes of 70 (S1 File) and 120 nt (results with the longer window size were unchanged [data not shown], this 70 nt window was used in subsequent analyses). RNA structural metrics were calculated for windows using the RNAfold algorithm [40] using the Turner energy model [41, 42] at 37°C. Z-score calculations were performed using the following equation (adapted from the approach of [13]):

$$z-score=({\Delta G{}^{\circ}}_{native}-{\bar{\Delta G{}^{\circ}}}_{random})/\sigma$$ (Eq 1)

Here, ΔG°_*native*_ is the native sequence minimum free energy (MFE) predicted by RNAfold. ${\bar{\Delta G{}^{\circ}}}_{random}$ is the average MFE predicted for 100X mononucleotide randomized sequences. The standard deviation, σ, is calculated across all sequences. The other calculated data are: the P-value, which measures the fraction of random sequences that are more stable than native in the z-score calculation (this acts as a quality control measure for the z-score); the MFE ΔG°, which measures the thermodynamic stability of RNA secondary structure formation; the MFE base pairs that generate the MFE ΔG°, which are output in “dot-bracket" notation; the ensemble diversity (ED), which provides an estimate of the structural diversity in the RNA conformational ensemble based on the calculation of the RNA partition function and comparisons between Boltzmann-weighted conformations that measure the number of base pairs that are different between each (low ED indicates a single dominant conformation while high ED indicates multiple conformations or a lack of structure); the fraction of the (f)MFE in the ensemble, which estimates the contribution of the MFE conformation to the ensemble; the ensemble centroid structure, which is the conformation most similar to others in the ensemble; and the nt frequencies and GC percentages.

ScanFold-Scan prediction windows were next analyzed using the program ScanFold-Fold to deduce consensus motifs weighted by the z-score. The ScanFold-Fold method is detailed in [12]. Resulting output consisted of a list of all base pairing partners predicted for each nucleotide of the *MYC* mRNA (S8 File) and a list of the most favorable base pairing arrangements when weighting by z-score (S9 File). From the latter, base pairs which contributed to consistently negative z-scores (i.e. bps with average z-scores < -1 from S9 File) were used as constraints in an RNAfold prediction on the entire mRNA under the additional constraint of a maximum bp distance of 300 nt. Base pairs that extended ScanFold-Fold helices were identified and used to generate the final motif models (S2 File). For visualizing the results of modeling, 2D rendering were generated using VARNA [43] and figures were produced with Adobe Illustrator. The statistical analyses of global metrics (S1–S3 Tables) were undertaken using spreadsheet equations and box and whisker plots (S1 Fig) were generated using BoxPlotR [44].

For the analysis and comparison of conservation of ScanFold-Fold motifs across *MYC*, homologous mRNAs for 14 representative vertebrates from different clades (e.g. primates down to amphibians) were obtained from the NCBI RefSeq RNA database [45]. This database was also queried using BLAST [46] to deduce homologs for the “short” *MYC* 5' and 3' UTR sequences. Alignments for the mRNA (S3 File) and UTRs (S4 and S5 Files) were performed using MAFFT [47], implementing the MAFFT-E-INS-i and MAFFT-G-INS-i strategies, respectively [48].

A global model for the short 3' UTR (defined/used in a previous study of miRNA targeting [19]) was generated by constraining base pairs from Motif 17 and refolding the remaining sequence using RNAfold [40]. A consensus secondary structure for the short *MYC* 3' UTR was predicted (S3 Fig) using RNAalifold [20] with the 3' UTR alignment (S5 File) as input.

### Experimental analyses

#### Cell culture

HeLa cells (including stably transduced HeLa cells) were incubated at 37°C and 5% CO_2_ and maintained in DMEM supplemented with 10% FBS, penicillin and streptomycin, and L-glutamine. Cells were passaged at 60–100% confluence and used between 3–40 passages.

#### Transduction of HeLa cells for HNRNPL knockdown and western validation

Reduced expression of HNRNPL in HeLa cells was accomplished using lentivirus transduction. Lenti-X cells (Clontech Laboratories) in 6-well dishes (VWR) were co-transfected with 12 μg GIPZ-based vector DNA (Dharmacon), 6 μg psPAX2, and 3 μg pMD2.G packaging vectors (Addgene) using the CaPO_4_ method. The GIPZ shHNRNPL-targeting vector was purchased from Dharmacon (RHS4531-EG3191; clone V2LHS_132169). The GIPZ control vector was generated by cloning the shRNA from TRIPZ non-silencing control (RHS4743; Dharmacon) via XhoI and MluI. Medium was changed 4–6 h post transfection. Supernatant was harvested 48h after transfection and filtered using PVDF (0.45 μm; Millipore Sigma). Virus was freshly prepared for each use. HeLa cells were seeded at 2.5 × 10^5^ cells per well in a 6-well dish 24h prior to transduction. Viral supernatant with 7.5 μg/ml polybrene (Sigma Aldrich) incubated with cells for 4h prior to the addition of two times the volume fresh medium for 48 h incubation. Cells were then trypsinized, replated into a 10 cm dish, and puromycin (1 μg/ml) selection was applied for 2 days followed by maintenance dosing (0.5 μg/ml) during subsequent culture.

Transduced HeLa cells (control and KD) were lysed in RIPA buffer (50 mM TrisHCl, pH 8.0; 1 mM EDTA; 150 mM NaCl; 100 μM Na_3_VO_4_; 1.0% NP-40; 1.0% sodium deoxycholate; 0.1% SDS) with HALT protease inhibitor (ThermoFisher). Total protein concentrations were determined via BCA assay (ThermoFisher). One μg total protein per sample was loaded on a 4–20% mini-PROTEAN gel (Bio-Rad) for PAGE and subsequently transferred to a PVDF membrane using a submarine blotter. The membrane was blocked for 1 h in tris-buffered saline with Tween-20 (TBST) containing 5% non-fat dry milk before a 1 h incubation with primary antibodies that targeted HNRNPL (1 μg/ml; BETHYL; cat# A303-895A) or HNRNPA1 (0.2 μg/ml; Santa Cruz; cat# sc-56700; loading control). After incubation with rabbit- and mouse-derived secondary antibodies conjugated to HRP (1:2000 dilution; Invitrogen; cat# G-21234 and 62–6520 respectively) for 40 min, Pierce ECL Western blotting substrate (ThermoFisher) was used for visualization on a Fotodyne gel imager (2x2 binning, 300 gain, 10-minute exposure). Densitometry was determined in Adobe Photoshop and analyzed with Microsoft Excel whereby the intensity of the HNRNPL band was normalized to the intensity of the corresponding HNRNPA1 band prior to comparing the KD to the control signal.

#### Luciferase vectors

For our experiments, two luciferase plasmid vector backbones were used. Both the transfection control vector, pIS0, which encodes *Firefly* (FF) luciferase, and the experimental vector, pIS2, which encodes *Renilla* (RL) luciferase, were gifts from David Bartel (Addgene plasmid # 12178; http://n2t.net/addgene:12178; RRID:Addgene_12178) and (Addgene plasmid # 12177; http://n2t.net/addgene:12177; RRID:Addgene_12177). The pcDNA3.1-miR34a vector was a gift from Heidi Schwarzenbach (Addgene plasmid # 78125; http://n2t.net/addgene:78125; RRID:Addgene 78125).

To test the post-transcriptional regulation of ScanFold-Fold predicted motifs, the Motif 17 sequence, along with 27 nt upstream and 11 nt downstream were incorporated into the 3' UTR of pIS2 to generate pIS2-M17. Mutants that destabilize (pIS2-AS1) or stabilize (pIS2-LS1) the structure present in pIS2-M17 were generated. For pIS2-AS1, six mutations were incorporated that disrupt canonical base pairing in the first conserved hairpin. To generate pIS2-LS1, three mutations and one base deletion were introduced in the bulge on the upstream side of the first conserved hairpin. The pIS2-LS1-CM vector had three base mutations introduced which reintroduce a bulge on the lower part of the first highly conserved hairpin of Motif 17 by disrupting the binding introduced by the pIS2-LS1 mutations. Mutations that destabilize or stabilize Motif 17 were predicted using the ΔΔG metric as a measure of miRNA site accessibility [26]. 70 nt upstream and 70 nt downstream of the miRNA target site were included in our ΔΔG calculations [26].

The sequences for pIS2-M17, pIS2-AS1, pIS2-LS1, and pIS2-LS1-CM were ordered as gBlocks from IDT and cloned using AgeI (5') and Spe1 (3') restriction sites (sequences in S5 Table). Insertion of experimental sequences into the 3' UTR of pIS2 required double restriction enzyme digest (using AgeI and SpeI from NEB) of both the gBlock and pIS2; following digestion, fragment and vector DNA were purified (Zymo DNA Clean and Concentrator kit), ligated (T4 Ligase from ThermoFischer), and transformed into DH5α-T1 competent cells using standard procedures. Carbenicillin selected colonies were cultured and plasmids were extracted (Qiaprep kit) and sequenced using an Applied Biosystems 3730xl DNA Analyzer.

#### Dual luciferase assay

Dual luciferase assays followed recommendations of an established method [49]. In brief, the pIS0 vector (FF) is transfected at constant levels across all samples to serve as an internal control to which RL luciferase expression is normalized. All samples were run as at least biological triplicates. HeLa cells were counted using a hemocytometer and plated in a 24-well dish at a density of 50,000 cells per well. After 48h, cells were transfected using Lipofectamine 3000 (ThermoFischer) with 500 nanograms total plasmid DNA at a 1:1:8 ratio (pIS0:pIS2-based:pcDNA3.1-miR34a). However, the assay conducted with the pIS2-LS1-CM vector and the HNRNPL KD assay were plated at 125,000 cells per well and transfected after 24h. Twenty-four hours after transfection, cells were trypsinized, resuspended, and split into each of a 24-well plate for RNA analysis (1 ml) and a 96-well plate for the dual luciferase assay (0.2 ml). After another 24h incubation, cells in the 96-well dish were lysed, and luciferase activity was measured using Promega’s Dual Luciferase Reagent Assay kit on a Biotek Synergy 2 plate reader with a collection time of 10 seconds. Relative response ratios (RRR), the ratio of RL to FF relative light units (RLUs), were calculated for each sample and then normalized to the empty, unregulated pIS2 RRR. Cells from the 24-well plate were placed in TRIzol (ThermoFisher) and either stored at -80°C or immediately processed as below.

#### RNA processing and qPCR analysis

Cellular RNA was purified from samples in TRIzol using Zymo’s Direct-Zol RNA Miniprep kit. Purified RNA was then Dnase I treated (NEB) for 2h at 37°C and the resulting DNase-treated RNA was purified with Zymo’s RNA Clean and Concentrator kit. Reverse transcription was done using 1 μg of purified RNA, random hexamers, and Superscript III (ThermoFisher).

Relative abundance of RL transcripts across samples were measured by qPCR, performed using PowerUp SYBR Green Master Mix on 1% cDNA input on an Applied Biosystems QuantStudio 3 instrument (ThermoFisher). Data were analyzed using the ΔΔCt method (S6 File), where the relative abundance of RL transcripts in the samples were determined using the FF transcript as the reference gene. Translational efficiencies (TE), a normalization metric (RRR/2^[-ΔΔCt^_RL_^]^), were calculated for each sample. Primers used in qPCR were: RL FWD 5'-GGAATTATAATGCTTATCTACGTGC-3'; RL REV 5'-CTTGCGAAAAATGAAGACCTTTTAC-3'; FF FWD 5'-CTCACTGAGACTACATCAGC-3'; and FF REV 5'-TCCAGATCCACAACCTTCGC-3'.

All data are available in the Supplemental Information. ScanFold-Scan and ScanFold-Fold are available for download from GitHub: https://github.com/moss-lab/ScanFold. RNAfold and RNAalifold are both bundled within the ViennaRNA package [40]: available at: https://www.tbi.univie.ac.at/RNA/.

## Supporting information

S1 Fig Whisker plots considering windows spanning different regions of the long MYC UTRs. Plots were generated using the BoxPlotR tool [44]. Center lines show the medians; box limits indicate the 25^th^ and 75^th^ percentiles as determined by R software; whiskers extend 1.5 times the interquartile range from the 25^th^ and 75^th^ percentiles, outliers are represented by dots.
(EPS)

S2 Fig Short MYC 5' UTR model. The main Figure shows the model previously determined using in vitro chemical mapping data [6]. The location of ScanFold-Fold Motif 8 is annotated on Domain 1, while the Motif 9 alternative to Domain 2 is shown in the insert. Base pair conservation shading indicated in the key, and data are taken from a comparison of 50 RefSeq mRNA vertebrate alignment (S4 File). To the right is the secondary structure of Motif 9 (Fig 1) annotated with data from a previous chemical mapping study of the MYC IRES [6]. Large and small arrows are for strong and weak chemical reactivities (reagents sensitive to single-stranded RNA); when arrows are in red, they conflict with the Motif 9 model (e.g. modification site occurs at a nt that is Watson-Crick paired within a helix formed by canonical Watson-Crick pairs). Circled nt indicate AMV reverse transcriptase (RT) pausing sites, which indicate structured regions.
(EPS)

S3 Fig RNAalifold consensus secondary structure for the MAFFT alignment of vertebrate *MYC* RefSeq short 3' UTRs (S5 File). Base pairs are colored by their conservation and the observation of different pairing types (see key on figure). Circled bases indicate structure-preserving consistent and compensatory base mutations. Lines in the consensus sequence indicated that gaps are predominate at the aligned position.
(EPS)

S4 Fig Annotations of miRNA binding sites on ScanFold-Fold predicted motifs. A) Shows miRNA sequences above the “dot-bracket” structure of Motif 17 (matched brackets indicated base pairs). Seed sites and the complements on Motif 17 are colored. B) Shows miRNA seed binding sites annotated on the 2D model of Motif 17. C) Shows base-pairing between miR-24 and the 2D model of Motif 18. D) Shows base-pairing between miR-24 and the 2D model of Motif 15.
(EPS)

S1 Table Correlation between metrics. Correlations between metrics for all scanning windows (raw data in S1 File). For each, correlation coefficients are reported, with values above 0.5 in bold.
(DOCX)

S2 Table Mean values of metrics for each mRNA region. For each region of the mRNA, metrics from all overlapping windows were averaged. Here we defined regions based on the coding sequence position described for NM_002467.5 (nt 1161 to 2525). The windows used for the analysis can be found in S1 File and were defined as follows: 5' UTR–windows 1 to 1091; 5' junction–windows 1092 to 1161; ORF–windows 1162 to 2456; 3' junction–windows 2457 to 2525; 3'UTR–windows 2526 to 4449.
(DOCX)

S3 Table Matrix of t-test p-values calculated for mean values of metrics between each mRNA region. This matrix holds the p-values of a two-tailed t-test assuming unequal variance between the corresponding regions. P-values greater than 0.01 are bolded.
(DOCX)

S4 Table Percentage of Motif base pairs predicted in the unconstrained global model of MYC mRNA folding (S2 File). (DOCX)

S5 Table gBlock sequences used for generation of pIS2-M17, pIS2-AS1, pIS2-LS1, and pIS2-LS1-CM. Base mutations, compared to WT pIS2-M17 sequence, are shown in bold.
(DOCX)

S1 File Excel document containing ScanFold-Scan results. Columns A–O contain: the i and j coordinates for each mRNA sequence; the minimum free energy (MFE) ΔG in kcal/mol; the z-score, calculated from Eq 1 (as described in the Materials and methods section); the P-value, in the z-score calculation (acts as a quality control); the ensemble diversity (ED); the fraction (f)MFE; the sequence of the window fragment; the MFE base pairs, in dot-bracket notation (pairs are matched brackets); the ensemble centroid base pairs; the frequencies of A, G, C and U; then, finally, the GC%.
(XLSX)

S2 File Dot-bracket structures. ScanFold-Fold predicted pairs for the short MYC mRNA (NM_002467.5) at -1 and -2 cutoff values, followed by the “filled in” motifs that were refolded with RNAfold. This is followed by the model structure of the 5' UTR based on previous studies as well as the constrained RNAfold model for the 3' UTR.
(TXT)

S3 File MAFFT alignment of select vertebrate MYC RefSeq mRNAs. (FA)

S4 File MYC 5’ UTR sequence alignments. (FASTA)

S5 File MYC 3’ UTR sequence alignments. (FASTA)

S6 File Raw and processed RLU and qPCR data used for generation of Fig 3B and 3C. (XLSX)

S7 File RBPMap results for Motif 17. (TXT)

S8 File ScanFold-Fold log file for all base pairs. (TXT)

S9 File ScanFold-Fold log file—Final Motif base pairs. (TXT)
