# Supplementary material for: RNA structural analysis of the MYC mRNA reveals conserved motifs that affect gene expression
Source: PLoS One. 2019 Jun 17;14(6):e0213758. doi: 10.1371/journal.pone.0213758 (PMC6576772; doi:10.1371/journal.pone.0213758)
Supplement: S1 Table — For each, correlation coefficients are reported, with values above 0.5 in bold. (DOCX) [file pone.0213758.s005.docx]

|  | **Native_dG** | **Z-score** | **P-value** | **ED** |
| --- | --- | --- | --- | --- |
| **Native_dG** | 1.000 |  |  |  |
| **Z-score** | 0.396 | 1.000 |  |  |
| **P-value** | 0.420 | **0.933** | 1.000 |  |
| **ED** | 0.067 | **0.572** | **0.545** | 1.000 |

**S1 Table. Correlation between metrics.** Correlations between metrics for all scanning windows (raw data in S1 File). For each, correlation coefficients are reported, with values above 0.5 in bold.
