# Supplementary material for: RNA structural analysis of the MYC mRNA reveals conserved motifs that affect gene expression
Source: PLoS One. 2019 Jun 17;14(6):e0213758. doi: 10.1371/journal.pone.0213758 (PMC6576772; doi:10.1371/journal.pone.0213758)
Supplement: S2 Table — For each region of the mRNA, metrics from all overlapping windows were averaged. Here we defined regions based on the coding sequence position described for NM_002467.5 (nt 1161 to 2525). The windows used for the analysis can be found in S1 File and were defined as follows: 5' UTR–windows 1 to 1091; 5' junction–windows 1092 to 1161; ORF–windows 1162 to 2456; 3' junction–windows 2457 to 2525; 3'UTR–windows 2526 to 4449. (DOCX) [file pone.0213758.s006.docx]

|  | **5' UTR** | **5' junction** | **ORF** | **3' junction** | **3' UTR** |
| --- | --- | --- | --- | --- | --- |
| **Native_dG** | -18.93 | -17.69 | -16.79 | -9.49 | -10.54 |
| **ED** | 11.90 | 15.17 | 12.59 | 14.81 | 12.15 |
| **Z-score** | -0.54 | -0.17 | -0.29 | -0.08 | -0.45 |

**S2 Table. Mean values of metrics for each mRNA region.** For each region of the mRNA, metrics from all overlapping windows were averaged. Here we defined regions based on the coding sequence position described for NM_002467.5 (nt 1161 to 2525). The windows used for the analysis can be found in S1 File and were defined as follows: 5' UTR – windows 1 to 1091; 5' junction – windows 1092 to 1161; ORF – windows 1162 to 2456; 3' junction – windows 2457 to 2525; 3'UTR – windows 2526 to 4449.
