# Supplementary material for: RNA structural analysis of the MYC mRNA reveals conserved motifs that affect gene expression
Source: PLoS One. 2019 Jun 17;14(6):e0213758. doi: 10.1371/journal.pone.0213758 (PMC6576772; doi:10.1371/journal.pone.0213758)
Supplement: S3 Table — This matrix holds the p-values of a two-tailed t-test assuming unequal variance between the corresponding regions. P-values greater than 0.01 are bolded. (DOCX) [file pone.0213758.s007.docx]

| **Native_dG** | **5'UTR** | **3'UTR** | **5'Junction** | **3'Junction** | **ORF** |
| --- | --- | --- | --- | --- | --- |
| **5'UTR** | - | - | - | - | - |
| **3'UTR** | 3.99E-159 | - | - | - | - |
| **5'Junction** | 8.67E-03 | 4.76E-15 | - | - | - |
| **3'Junction** | 1.23E-72 | 3.63E-04 | 2.01E-34 | - | - |
| **ORF** | 3.06E-13 | 8.28E-205 | 0.0352 | 3.46E-48 | - |
|  |  |  |  |  |  |
| **ED** | **5'UTR** | **3'UTR** | **5'Junction** | **3'Junction** | **ORF** |
| **5'UTR** | - | - | - | - | - |
| **3'UTR** | **0.266** | - | - | - | - |
| **5'Junction** | 1.92E-04 | 1.40E-07 | - | - | - |
| **3'Junction** | 2.34E-07 | 9.69E-07 | **0.632** | - | - |
| **ORF** | 0.00716 | **0.0487** | 0.00104 | 2.60E-05 | - |
|  |  |  |  |  |  |
| **z-score** | **5'UTR** | **3'UTR** | **5'Junction** | **3'Junction** | **ORF** |
| **5'UTR** | - | - | - | - | - |
| **3'UTR** | **0.0287** | - | - | - | - |
| **5'Junction** | 0.0068 | 4.84E-04 | - | - | - |
| **3'Junction** | 2.7E-07 | 1.22E-05 | **0.574** | - | - |
| **ORF** | 1.1E-08 | 2.29E-04 | 0.001 | 0.00604 | - |

**S3 Table. Matrix of t-test p-values calculated for mean values of metrics between each mRNA region.** This matrix holds the p-values of a two-tailed t-test assuming unequal variance between the corresponding regions. P-values greater than 0.01 are bolded.
