# Supplementary material for: RNA structural analysis of the MYC mRNA reveals conserved motifs that affect gene expression
Source: PLoS One. 2019 Jun 17;14(6):e0213758. doi: 10.1371/journal.pone.0213758 (PMC6576772; doi:10.1371/journal.pone.0213758)
Supplement: S4 Table — (DOCX) [file pone.0213758.s008.docx]

| **Motif** | **% bp predicted in the global fold** |
| --- | --- |
| Motif 1 | 0% |
| Motif 2 | 0% |
| Motif 3 | 0% |
| Motif 4 | 0% |
| Motif 5 | 60% |
| Motif 6 | 54% |
| Motif 7 | 64% |
| Motif 8 | 45% |
| Motif 9 | 100% |
| Motif 10 | 0% |
| Motif 11 | 100% |
| Motif 12 | 73% |
| Motif 13 | 46% |
| Motif 14 | 81% |
| Motif 15 | 54% |
| Motif 16 | 0% |
| Motif 17 | 91% |
| Motif 18 | 100% |
| Motif 19 | 45% |
| Motif 20 | 0% |
| Motif 21 | 73% |
| Motif 22 | 88% |
| Motif 23 | 80% |
| Motif 24 | 0% |
| Motif 25 | 94% |
| Motif 26 | 53% |
| Motif 27 | 100% |
| Motif 28 | 100% |
| Motif 29 | 100% |
| Motif 30 | 100% |

**S4 Table. Percentage of Motif base pairs predicted in the unconstrained global model of MYC mRNA folding (S2 File).**
