# Supplementary material for: RNA structural analysis of the MYC mRNA reveals conserved motifs that affect gene expression
Source: PLoS One. 2019 Jun 17;14(6):e0213758. doi: 10.1371/journal.pone.0213758 (PMC6576772; doi:10.1371/journal.pone.0213758)
Supplement: S5 Table — Base mutations, compared to WT pIS2-M17 sequence, are shown in bold. (DOCX) [file pone.0213758.s009.docx]

**S5 Table. gBlock sequences used for generation of pIS2-M17, pIS2-AS1, pIS2-LS1, and pIS2-LS1-CM**. Base mutations, compared to WT pIS2-M17 sequence, are shown in bold.
